# Supplementary material for: MogR Is a Ubiquitous Transcriptional Repressor Affecting Motility, Biofilm Formation and Virulence in Bacillus thuringiensis
Source: Front Microbiol. 2020 Dec 23;11:610650. doi: 10.3389/fmicb.2020.610650 (PMC7793685; doi:10.3389/fmicb.2020.610650)
Supplement: Supplementary file 1 [file Data_Sheet_1.pdf]

Supplementary Material

MogR is a ubiquitous transcriptional repressor affecting motility, biofilm formation and virulence in *Bacillus thuringiensis*

Veronika Smith, Malin Josefsen, Toril Lindbäck, Ida K. Hegna, Sarah Finke, Nicolas J. Tourasse, Christina Nielsen-LeRoux, Ole Andreas Økstad, Annette Fagerlund

1 Supplementary Figures

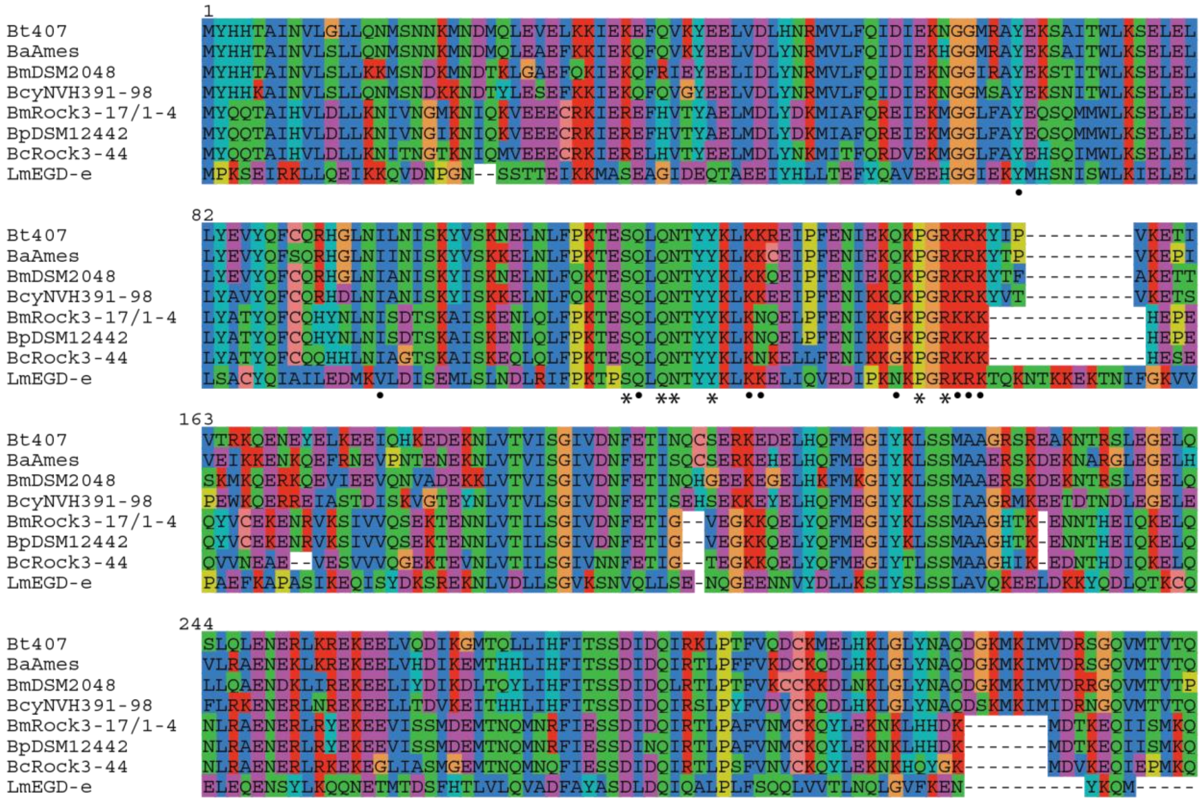

Supplementary Figure 1. Multiple sequence alignment of MogR protein sequences. MogR sequences were extracted from *L. monocytogenes* strain EGD-e (locus tag: lmo0674), *B. cytotoxicus* NVH 391/98 (Bcer98_1372), and *B. thuringiensis* 407 (BTB_RS08390), in addition to the following predicted non-motile strains: The group I strains *B. cereus* Rock 3-44 (bcere0022_13670), *B. pseudomycoides* DSM 12442 (bpmyx0001_15070) and *B. mycoides* Rock 3-17 and Rock 1-4 (bmyco0002_14720 and bmyco0003_14260, which are identical), the *B. anthracis* Ames strain (BA_1692), and the group VI strain *B. mycoides* DSM 2048 (bmyco0001_14970). The annotated start site of the MogR protein from *B. mycoides* DSM 2048 was adjusted to fit the start site predicted by EasyGene (Larsen and Krogh, 2003). The N-terminal amino acid sequence MLSGSEYK of MogR from the group VI *B. mycoides* strains and *B. pseudomycoides* DSM 12442 were trimmed from the alignment. Residues making DNA backbone contacts and those making base specific DNA contacts, as determined for *L. monocytogenes* MogR by Shen et al. (2009), are marked with a solid dot and a star, respectively.

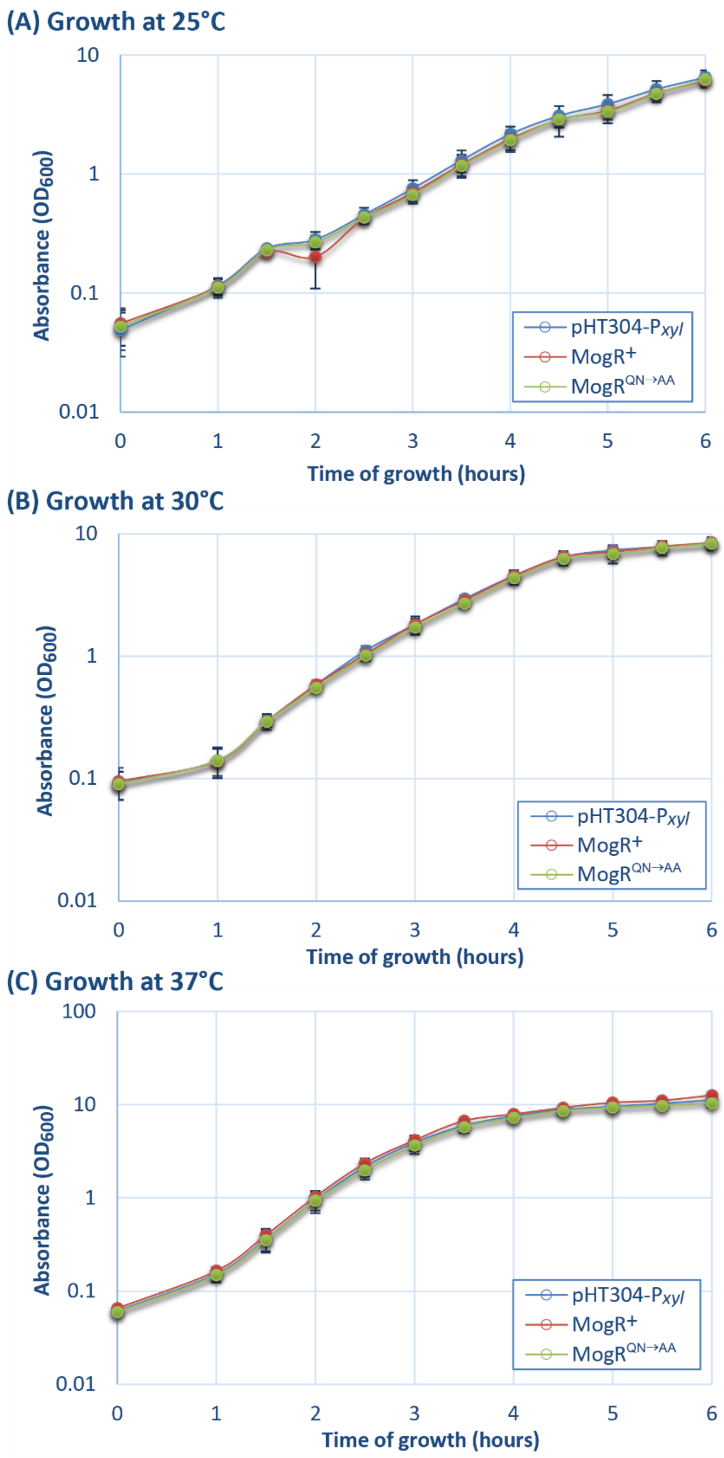

Supplementary Figure 2. Growth curves at A) 25 °C, B) 30 °C and C) 37 °C. Assays were conducted using *B. thuringiensis* 407 pHT304-P$_{xyl}$, an empty vector control strain (pHT304-P$_{xyl}$); *B. thuringiensis* 407 pHT304-P$_{xyl}$-*mogR* (MogR^{+}), a strain overexpressing MogR from the pHT304-P$_{xyl}$ plasmid vector; and *B. thuringiensis* 407 pHT304-P$_{xyl}$-*mogR*$^{QN\rightarrow AA}$ (MogR$^{QN\rightarrow AA}$), a strain overexpressing a mutated form of MogR from the pHT304-P$_{xyl}$ vector.

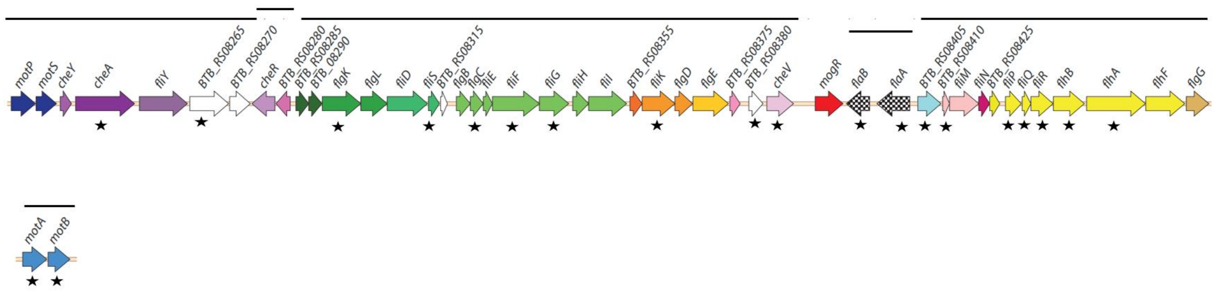

Supplementary Figure 3. Graphic representation of genes in the motility loci from *B. thuringiensis* 407. Solid lines above genes indicate operon structures, predicted from RNA-Seq data obtained from *B. cereus* strains ATCC 10987 and ATCC 14579 (Kristoffersen et al., 2012). Annotated genes are indicated with gene names positioned above the arrows. Genes affected by MogR overexpression are indicated by an asterisk.

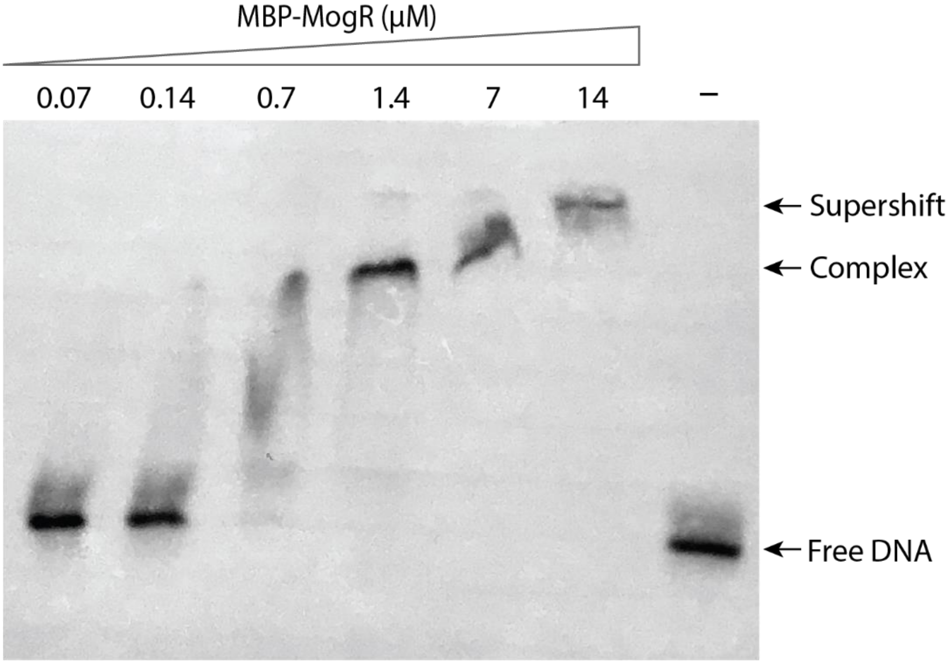

Supplementary Figure 4. EMSA showing titration of MogR protein. The experiment was carried out with biotin-labeled *fla* promoter region DNA and increasing concentrations of purified MBP-MogR fusion protein. A concentration of 1.4 µM is equivalent to 1 µg of the 37 kDa MBP-MogR protein in the binding reaction. The positions of free labelled DNA probe, shifted protein-DNA complex and supershifted protein-DNA complex are indicated. See Figure 5A for location of primers used for generating the *fla* promoter probe.

2 Supplementary Tables

Supplementary Table 1. Reciprocal best BLAST hit analysis for motility proteins.

Genomes of the following strains were subject to analyses: *B. thuringiensis* 407, *L. monocytogenes* EGD-e, and *B. subtilis* 168.

		Locus tags and seqIDs. Reference proteins are in shown in bold			Reciprocal best Blast hits*		
Function / gene product	gene	Bt407	EGDe	Bs168	Bt407 vs EGDe	Bt407 vs Bs168	EGDe vs Bs168
Flagellar motor protein MotP (Na$^+$-coupled stator)	*motP*	BTB_RS08240		BSU29730_ ytxD	no	yes (37%/92%)	no
Flagellar motor protein MotS (Na$^+$-coupled stator)	*motS*	BTB_RS08245		BSU29720_ ytxE	no	yes (39%/95%)	no
Chemotaxis protein, response regulator CheY	*cheY*	BTB_RS08250	lmo0691_cheY	BSU16330_cheY	yes (75%/98%)	yes (68%/100%)	yes (69%/95%)
Chemotaxis protein, histidine kinase CheA	*cheA*	BTB_RS08255	lmo0692_cheA	BSU16430_cheA	yes (56%/105%)	yes (44%7103%)	yes (45%/103%)
Flagellar motor switch FliY	*fliY*	BTB_RS08260	lmo0700	BSU16320_fliY	yes (42%/99%)	yes (35%/76%)	yes (38%/65%)
hypothetical protein		BTB_RS08265			no	no	
hypothetical protein		BTB_RS08270			no	no	
Chemotaxis protein, methyltransferase CheR	*cheR*	BTB_RS08275	lmo0683	BSU22720_cheR	yes (44%/98%)	yes (48%/98%)	yes (47%/98%)
hypothetical protein		BTB_RS08280	lmo0687		yes (43%/100%)	no	no
Protein of unknown function (DUF327)		BTB_RS08285	lmo0703	BSU00300_yaaR	yes (48%/93%)	yes (25%/89%)	yes (24%/55%)
FlgN-like superfamily protein		BTB_RS08290			no	No	
Flagellar hook-associated protein FlgK	*flgK*	BTB_RS08295_flgK	lmo0705_flgK	BSU35410_flgK	yes (35%/62%)	yes (27%/47%)	yes (22%/106%)
Flagellar hook-associated protein FlgL	*flgL*	BTB_RS08300_flgL	lmo0706_flgL	BSU35400_flgL	yes (30%/101%)	yes (27%/93%)	yes (28%/69%)
Flagellar hook-associated protein FliD	*fliD*	BTB_RS08305_fliD	lmo0707_fliD	BSU35340_fliD	yes (28%/97%)	yes (24%/81%)	yes (24%/107%)
Flagellar protein FliS	*fliS*	BTB_RS08310	lmo0708	BSU35330_fliS	yes (54%/97%)	no	yes (21%/96%)
hypothetical protein		BTB_RS08315	lmo0709		no**	no	no
Flagellar basal-body rod protein FlgB	*flgB*	BTB_RS08320_flgB	lmo0710_flgB	BSU16180_flgB	yes (42%/101%)	yes (27%/83%)	yes (31%/95%)
Flagellar basal-body rod protein FlgC	*flgC*	BTB_RS08325	lmo0711_flgC	BSU16190_flgC	yes (58%/100%)	yes (44%/103%)	yes (41%/103%)
flagellar hook-basal body protein FliE	*fliE*	BTB_RS08330_fliE	lmo0712_fliE	BSU16200_fliE	yes (52%/84%)	No	yes (26%/71%)
Flagellar MS-ring protein FliF	*fliF*	BTB_RS08335	lmo0713_fliF	BSU16210_fliF	yes (39%/97%)	yes (22%/99%)	yes (24%/77%)
Flagellar motor switch protein FliG	*fliG*	BTB_RS08340	lmo0714_fliG	BSU16220_fliG	yes (46%/95%)	yes (34%/99%)	yes (32%/96%)
Flagellar assembly protein H	*fliH*	BTB_RS08345_fliH	lmo0715_fliH		yes (37%/104%)	no	no
Flagellum-specific ATP synthase FliI	*fliI*	BTB_RS08350_fliI	lmo0716_fliI	BSU16240_fliI	yes (70%/99%)	yes (44%/96%)	yes (44%/97%)
hypothetical protein		BTB_RS08355	lmo0694		yes (34%/99%)	no	no
hypothetical protein with C-terminal domain of FliK		BTB_RS08360	lmo0695		yes (34%/27%)	no	no
Flagellar basal body rod modification protein FlgD	*flgD*	BTB_RS08365_flgD	lmo0696_flgD	BSU16280_flgD	yes (45%/78%)	yes (31%/60%)	yes (33%/96%)
Fagellar hook-basal body protein, FlgE/F/G	*flgE/F/G*	BTB_RS08370	lmo0697_flgE	BSU16290_flgG	yes (47%/102%)	yes (37%/40%)	yes (39%/50%)
Protein of unknown function DUF3964		BTB_RS08375	lmo0684		yes (41%/101%)	no	no
flagellar motor switch FliG superfamily protein		BTB_RS08380			No	no	
Chemotaxis signal transduction protein CheV	*cheV*	BTB_RS08385	lmo0689	BSU14010_cheV	yes (64%/102%)	yes (49%/100%)	yes (49%/99%)
Motility gene repressor MogR	*mogR*	BTB_RS08390	lmo0674_mogR		yes (35%/89%)	no	no
flagellin	*fla/hag*	BTB_RS08395	lmo0690_flaA	BSU35360_hag	yes (33%/104%)	yes (30%/109%)	yes (39%/105%)
flagellin	*flaA*	BTB_RS08400			no	no	
Putative lytic murein transglycosylase		BTB_RS08405	lmo0717	BSU11570_yjbJ	yes (49%/82%)	yes (46%/69%)	yes (59%/56%)
Flagellar motor switch FliN	*fliN*	BTB_RS08410	lmo0698		yes (50%/95%)	no	no
Flagellar motor switch protein FliM	*fliM*	BTB_RS08415	lmo0699_fliM	BSU16310_fliM	yes (49%/98%)	yes (26%/98%)	yes (25%/101%)
Flagellar motor switch FliN	*fliN*	BTB_RS08420	lmo0693		yes (57%/70%)	no	no
Putative flagellar motor switch FliN		BTB_RS08425	lmo0675		yes (32%/97%)	no	no
Flagellar biosynthesis protein FliP	*fliP*	BTB_RS08430	lmo0676_fliP	BSU16350_fliP	yes (62%/84%)	yes (38%/93%)	yes (34%/89%)
Flagellar biosynthesis protein FliQ	*fliQ*	BTB_RS08435	lmo0677_fliQ	BSU16360_fliQ	yes (49%/98%)	yes (33%/93%)	yes (44%/60%)

Fflagellar biosynthesis protein FliR	*fliR*	BTB_RS08440	lmo0678_fliR	BSU16370_fliR	yes (47%/100%)	yes (24%/98%)	yes (24%/93%)
Flagellar biosynthesis protein FlhB	*flhB*	BTB_RS08445	lmo0679_flhB	BSU16380_flhB	yes (48%/100%)	yes (33%/97%)	yes (34%/98%)
Flagellar biosynthesis protein FlhA	*flhA*	BTB_RS08450	lmo0680_flhA	BSU16390_flhA	yes (65%/100%)	yes (37%/100%)	yes (36%/101%)
Flagellar biosynthesis regulator FlhF	*flhF*	BTB_RS08455_flhF	lmo0681	BSU16400_flhF	yes (33%/67%)	yes (29%/67%)	yes (34%/47%)
Flagellar basal-body rod protein FlgG	*flgG*	BTB_RS08460_flgG	lmo0682_flgG		yes (42%/98%)	no	no
Flagellar motor protein MotA (H$^+$-coupled stator)	*motA*	BTB_RS22910	lmo0685	BSU13690_motA	yes (43%/96%)	yes (51%/99%)	yes (49%/91%)
Flagellar motor protein MotB (H$^+$-coupled stator)	*motB*	BTB_RS22905_motB	lmo0686_motB	BSU13680_motB	yes (45%/97%)	yes (48%/95%)	yes (44%/97%)
Glycosyltransferase. In *L. monocytogenes*: Flagellin glycosyltransferase and MogR antirepressor GmaR**	*gmaR****	BTB_RS06215***	lmo0688	BSU21450_yolJ***	yes (33%/42%)	yes (27%/54%)	yes (27%/53%)
hypothetical protein			lmo0701		no		no
hypothetical protein			lmo0702		no		no
hypothetical protein			lmo0704		no		no
hypothetical protein			lmo0718		no		no
Flagellar assembly protein H	*fliH*			BSU16230_fliH		no	no
Flagellar export protein FliJ	*fliJ*			BSU16250_fliJ		no	no
flagellar motor switch protein FliG superfamily protein				BSU16260_ylxF		no	no
Flagellar hook-length control protein FliK	*fliK*			BSU16270_fliK		no	no
flagellar protein				BSU16299_ylzI		no	no
Flagellar basal body-associated protein FliL	*fliL*			BSU16300_fliL		no	no
Flagellar biosynthesis protein, FliO	*fliO*			BSU16340_fliZ		no	no
flagellum site-determining protein YlxH				BSU16410_ylxH		no	no
Chemotaxis protein, response regulator CheB	*cheB*			BSU16420_cheB		no	no
Chemotaxis protein CheW	*cheW*			BSU16440_cheW		no	no
CheY-P phosphatase CheC	*cheC*			BSU16450_cheC		no	no
Chemoreceptor glutamine deamidase CheD	*cheD*			BSU16460_cheD		no	no
RNA polymerase sigma-D factor	*sigD*			BSU16470_sigD		no	no
swarming motility protein SwrB	*swrB*			BSU16480_ylxL		no	no
Swarming motility protein SwrA	*swrA*			BSU35239-BSU35230_swrA****		no	no
ribosome hibernation promoting factor protein	*yvyD*	BTB_RS26450	lmo2511	BSU35310_yvyD	yes (63%/101%)	yes (67%/102%)	yes (60%/101%)
hypothetical protein				BSU35319_yvzG		no	no
flagellar protein FliT	*fliT*			BSU35320_fliT		no	no
FlaG family protein YvyC				BSU35350_yvyC		no	no
Carbon storage regulator CsrA	*csrA*			BSU35370_csrA		no	no
flagellar assembly factor FliW	*fliW*			BSU35380_yviF		no	no
hypothetical protein				BSU35390_yviE		no	no
FlgN-like family protein				BSU35420_yvyG		no	no
negative regulator of flagellin synthesis	*flgM*			BSU35430_flgM		no	no
Flagellar operon protein YvyF	*yvyF*			BSU35440_yvyF		no	no

* In parentheses: Percentage of identical matches in alignment / Percentage overlap of best hit, calculated as length of alignment relative to query protein length (both calculated as averages obtained from the forward and reciprocal blast)

** Score below threshold, bitscore 26.6 (87%/26%)

*** Although these proteins are reciprocal BLAST hits to *Lm* EGD-e GmaR, they are not considered true GmaR orthologs, as they lack the GmaR-specific C-terminal MogR-binding anti-repressor domain.

**** Laboratory strains of *B. subtilis* are known to contain an single “A” insertion mutation giving a frameshift in the *swrA* gene (McLoon et al., 2011). The protein used as query in this analysis was in-frame.

Supplementary Table 2: Primers used in RT-qPCR

Gene name	Locus tag	Forward primer (5'-3')	Reverse primer (5'-3')	Product size	E	r^2
gatB	BTB_RS21880	agctggtcgtgaagaccttg	cggcataacagcagtcatca	175 bp	1.93	0.9968
rpsU	BTB_RS21885	aagatcggtttctaaaactggtaca	tttcttgccgcttcagattt	102 bp	1.87	0.9957
udp	BTB_RS26495	actagagaaacttggaaatgatcg	gacgcttaattgcacggaac	101 bp	1.85	0.9992
mogR	BTB_RS08390	gggatgcgagcatatgaaaa	aatgtttaaaccgtgacgttgac	102 bp	1.95	0.9992
flaB	BTB_RS08395	ctgcgaacggtacaaattca	aactcagtcgtctcgccaat	100 bp	1.96	0.9983
flaA	BTB_RS08400	ccgtgcaacactaggtgcta	cgtcttcgatttgagaagca	104 bp	1.94	0.9983

E, PCR efficiency. r^2, square of Pearson's correlation coefficient calculated from serial dilutions.

Supplementary Table 3: Genome-wide gene expression analysis. Differentially regulated genes identified by microarray-based transcriptional profiling of the MogR overexpression strain relative to an empty vector control. All genes with FDR-corrected *p*-values <0.05 are listed (ordered by locus tag number).

Genes downregulated in the MogR overexpression strain:

**Locus tag in *B. thuringiensis* 407**	**Locus tag in *B. cereus* ATCC 14579**	**Predicted function**	**log_2 (fold change)**	***p*-value (FDR-corrected)**
BTB_RS01475	BC0294	10 kDa chaperonin	-1.06	0.00
BTB_RS01480	BC0295	60 kDa chaperonin	-0.58	0.05
BTB_RS02175	BC0422	Methyl-accepting chemotaxis protein (with upstream "off" c-di-GMP riboswitch)	-1.31	0.00
BTB_RS02990	BC0587	Acetyltransferase	-1.21	0.00
BTB_RS03005	BC0590	Uncharacterized conserved protein YjgD	-0.73	0.03
BTB_RS03040	BC0598	Helix-turn-helix domain protein/NprR	-0.89	0.00
BTB_RS03260	BC0643	Amino-acid permease rocC	-0.63	0.02
BTB_RS03410	BC0666	Immune inhibitor A	-1.03	0.00
BTB_RS03430	BC0670	Phospholipase C (PC-PLC)	-2.32	0.00
BTB_RS03435	BC0671	Sphingomyelinase C (Smase)	-1.47	0.01
BTB_RS04285	BC0791	Coenzyme A disulfide reductase Cdr	-1.54	0.00
BTB_RS04490	BC0834	Hypothetical protein	-1.95	0.00
BTB_RS04760	BC0888	N-acetylmuramoyl-L-alanine amidase CwlH	-1.50	0.00
BTB_RS05200	BC1000	Hypothetical protein	-1.01	0.00
BTB_RS05215	BC1003	Serine-protein kinase rsbW	-0.91	0.00
BTB_RS05220	BC1004	RNA polymerase sigma-B factor	-1.05	0.00
BTB_RS05375	BC1030	Hypothetical protein	-0.51	0.05
BTB_RS05580	BC1061	Hypothetical protein	-0.82	0.02
BTB_RS05760	BC1113	Sigma-M negative effector	-1.61	0.01
BTB_RS06105	BC1185	Dipeptide-binding protein DppE	-0.93	0.00
BTB_RS06140	BC1193	Oligoendopeptidase F	-2.39	0.00
BTB_RS06705	BC1316	Hypothetical protein	-0.66	0.03
BTB_RS07240	BC1424	Ferredoxin--nitrite reductase NirA	-1.62	0.00
BTB_RS07935	BC1570	Xanthine permease PbuX	-1.21	0.02
BTB_RS08255	BC1628	Chemotaxis protein, histidine kinase CheA	-1.80	0.00
BTB_RS08265	BC1630	Hypothetical protein	-0.92	0.03
BTB_RS08295	BC1636	Flagellar hook-associated protein 1, FlgK	-1.67	0.02
BTB_RS08310	BC1639	Flagellar (assembly) protein FliS	-0.68	0.00
BTB_RS08325	BC1642	Flagellar basal-body rod protein FlgC	-2.87	0.00
BTB_RS08335	BC1644	Flagellar MS-ring protein FliF	-2.31	0.00
BTB_RS08340	BC1645	Flagellar motor switch protein FliG	-1.09	0.00
BTB_RS08360	BC1649	Flagellar hook-length control protein FliK	-1.47	0.00
BTB_RS08380	BC1653	Hypothetical protein	-1.37	0.00
BTB_RS08385	BC1654	Chemotaxis signal transduction protein CheV	-1.80	0.00
BTB_RS08395	BC1656	Flagellin FlaB	-1.04	0.00
BTB_RS08400	BC1657	Flagellin FlaA	-3.45	0.00
BTB_RS08405	BC1660	Putative lytic murein transglycosylase	-0.75	0.00
BTB_RS08410	BC1661	Flagellar motor switch protein (fliN-homolog)	-0.99	0.00
BTB_RS08430	BC1665	Flagellar biosynthesis protein FliP	-1.86	0.02
BTB_RS08435	BC1666	Flagellar biosynthesis protein FliQ	-1.23	0.00
BTB_RS08440	BC1667	Flagellar biosynthesis protein FliR	-1.02	0.00
BTB_RS08445	BC1668	Flagellar biosynthesis protein FlhB	-1.60	0.00
BTB_RS08450	BC1669	Flagellar biosynthesis protein FlhA	-1.43	0.00
BTB_RS09080	BC1789	Transcriptional regulatory protein	-1.27	0.00
BTB_RS10150	BC1991	Putative murein endopeptidase	-0.80	0.00
BTB_RS10480	BC2056	Hypothetical protein	-0.74	0.02
BTB_RS10590	BC2076	Acetyltransferase	-1.47	0.00
BTB_RS12170	BC2379	Transcriptional regulator	-1.54	0.05
BTB_RS12675	BC2478	ABC transporter, ATP-binding protein	-3.19	0.00
BTB_RS12935	BC2529	Zn-dependent alcohol dehydrogenase	-1.97	0.00
BTB_RS13545	BC2659	Glutamate-rich protein grpB	-1.72	0.00
BTB_RS14470	BC2832	Putative aldehyde dehydrogenase DhaS	-2.10	0.00
BTB_RS14520	BC2842	Hypothetical protein	-0.73	0.00
BTB_RS14700	BC2881	Uncharacterized protein YhbF	-2.10	0.00
BTB_RS14970	BC2925	Nucleotidyltransferase	-2.27	0.03
BTB_RS15135	BC2959	Putative malate:quinone oxidoreductase Mqo	-0.67	0.00

BTB_RS15165	BC2964	Transcriptional regulator LsrR	-2.12	0.00
BTB_RS12545	BC3102	Hemolysin BL-binding component HblA	-1.13	0.02
BTB_RS12540	BC3103	Hbl component L1	-1.59	0.00
BTB_RS17315	BC3461	putative deacetylase YojG	-1.33	0.00
BTB_RS17995	BC3586	Dipeptide-binding protein DppE	-1.15	0.00
BTB_RS18825	BC3762	Serine protease, subtilase family Sfp	-0.84	0.02
BTB_RS19450	BC3835	Ribonuclease HII	-1.24	0.00
BTB_RS19615	BC3867	Putative coenzyme A biosynthesis bifunctional protein CoaBC	-1.12	0.00
BTB_RS20130	BC3966	Hypothetical protein	-2.57	0.02
BTB_RS20410	BC4020	Hypothetical protein	-0.98	0.02
BTB_RS21125	BC4154	Hypothetical protein	-1.47	0.00
BTB_RS21330	BC4198	Xaa-Pro dipeptidase PepQ	-0.51	0.01
BTB_RS21660	BC4260	Glucokinase	-1.85	0.01
BTB_RS21815	BC4293	Putative phosphotransferase	-0.71	0.04
BTB_RS21885	BC4307	30S ribosomal protein S21	-0.81	0.00
BTB_RS21915	BC4313	protein GrpE	-0.75	0.00
BTB_RS21920	BC4314	Heat-inducible transcription repressor hrcA	-0.67	0.01
BTB_RS22300	BC4389	Helicase, RecD/TraA	-1.53	0.00
BTB_RS22905	BC4512	Flagellar motor protein MotB (H+-coupled stator)	-3.01	0.01
BTB_RS22910	BC4513	Flagellar motor protein MotA (H+-coupled stator)	-1.74	0.00
BTB_RS23315	BC4594	Citrate synthase 2	-0.63	0.00
BTB_RS23780	BC4683	putative ribosomal N-acetyltransferase YdaF	-1.06	0.00
BTB_RS24165	BC4756	GDP-mannose-dependent alpha-mannosyltransferase MgtA	-1.58	0.00
BTB_RS24190	BC4762	Phosphoenolpyruvate carboxykinase	-1.26	0.02
BTB_RS22105	BC4861	hypothetical protein	-1.60	0.03
BTB_RS25940	BC5048	Ferritin	-1.66	0.00
BTB_RS26545	BC5211	putative permease IIC component YwbA	-1.23	0.02
BTB_RS26650	BC5228	glycolate permease GlcA	-0.84	0.00
BTB_RS26730	BC5243	hypothetical protein	-0.87	0.01
BTB_RS26945	BC5280	3R)-hydroxymyristoyl-[acyl-carrier-protein] dehydratase FabZ	-0.68	0.00
BTB_RS27450	BC5380	putative siderophore-binding lipoprotein YfiY	-0.84	0.02

Genes upregulated in the MogR overexpression strain:

**Locus tag in *B. thuringiensis* 407**	**Locus tag in Bc ATCC 14579**	**Predicted function**	**$\log_2$ (fold change)**	**p-value**
BTB_RS02515	BC0492	Pyruvate formate-lyase-activating enzyme PflA	0.92	0.00
BTB_RS04390	BC0813	Cell wall-binding protein YocH	0.96	0.00
BTB_RS05575	BC1060	Collagen adhesion protein with upstream c-di-GMP "on" riboswitch CbpA	3.02	0.00
BTB_RS06050	BC1174	3-oxoacyl-[acyl-carrier-protein] synthase 2	0.96	0.00
BTB_RS06215	BC1208	Glycosyl transferase, group 2	1.23	0.00
BTB_RS06545	BC1283	SinI	1.08	0.05
BTB_RS07850	BC1553	Penicillin-binding protein 1A/1B	0.62	0.01
BTB_RS08390	BC1655	MogR	0.75	0.01
BTB_RS09115	BC1794	Dipeptide-binding protein DppE	2.01	0.00
BTB_RS09415	BC1853	Hypothetical protein	2.00	0.00
BTB_RS10950	BC2164	Isoleucine--tRNA ligase IleS	1.00	0.04
BTB_RS11420	BC2238	Hypothetical protein	1.95	0.00
BTB_RS12700	BC2483	Putative acyl-CoA dehydrogenase YngJ	1.81	0.00
BTB_RS20160	BC3972	Pyruvate dehydrogenase complex E1 component, beta subunit	0.71	0.01
BTB_RS20730	BC4083	Putative adenine permease PurP	0.73	0.03
BTB_RS20740	BC4085	Pyrimidine-nucleoside phosphorylase Pdp	0.80	0.02
BTB_RS20745	BC4086	Purine nucleoside phosphorylase 1	0.87	0.01
BTB_RS24745	BC4870	L-lactate dehydrogenase 2	0.66	0.02
BTB_RS26190	BC5138	Phosphoglycerate kinase, Pkk	0.83	0.00
BTB_RS26200	BC5141	Central glycolytic genes regulator, CggR	0.94	0.00
BTB_RS26300	BC5158	Integral membrane protein	1.58	0.02
BTB_RS26705	BC5238	Glycine betaine transporter OpuD	1.10	0.05
BTB_RS26935	BC5278	Putative capsular polysaccharide biosynthesis protein YwqC	0.71	0.02

Supplementary References

Kristoffersen, S.M., Haase, C., Weil, M.R., Passalacqua, K.D., Niazi, F., Hutchison, S.K., Desany, B., Kolstø, A.B., Tourasse, N.J., Read, T.D., and Økstad,O.A. (2012). Global mRNA decay analysis at single nucleotide resolution reveals segmental and positional degradation patterns in a Gram-positive bacterium. *Genome Biol.* 13, R30.

Larsen, T.S., and Krogh, A. (2003). EasyGene – a prokaryotic gene finder that ranks ORFs by statistical significance. *BMC Bioinform.* 4, 21.

McLoon, A.L., Guttenplan, S.B., Kearns, D.B., Kolter, R., and Losick, R. (2011). Tracing the domestication of a biofilm-forming bacterium. *J. Bacteriol.* 193, 2027-2034.

Shen, A., Higgins, D.E., and Panne, D. (2009). Recognition of AT-rich DNA binding sites by the MogR repressor. *Structure* 17, 769-777.
